# Case Report: Cor triatriatum sinister presenting as acute decompensated heart failure in an adolescent

**DOI:** 10.3389/fcvm.2026.1785372

**Published:** 2026-03-16

**Authors:** ZainEdeen Zyadah, Joyce Morcos, Alaa Alresheq, Salem K. Qupp, Ahmad Fatayer, Jahed Bushnaq

**Affiliations:** 1 Cardiology Interest Group of Jerusalem, Faculty of Medicine, Al-Quds University, Jerusalem, Palestine; 2 Department of Internal Medicine, Palestine Medical Complex, Ramallah, Palestine; 3 Internal Medicine, Washington University of Health and Science, San Pedro, Belize

**Keywords:** acute decompensated heart failure, cardiogenic pulmonary edema, congenital heart disease, cor triatriatum sinister, echocardiography

## Abstract

**Background:**

Cor Triatriatum sinister (CTS) is a rare congenital cardiac anomaly caused by a fibromuscular membrane subdividing the left atrium, potentially leading to impaired left ventricular inflow, pulmonary venous hypertension, and heart failure. Clinical presentation is highly variable and may mimic primary respiratory disease, resulting in delayed diagnosis, particularly in older children and adolescents.

**Case Summary:**

A 13-year-old previously healthy boy presented with acute hypoxic respiratory failure following one week of progressive exertional dyspnea. Initial findings of elevated jugular venous pressure and bilateral pulmonary congestion with low inflammatory markers suggested a cardiogenic etiology. Bedside lung ultrasonography demonstrated diffuse pulmonary oedema, and transthoracic echocardiography revealed a severely obstructive supramitral membrane consistent with cor Triatriatum sinister and secondary pulmonary hypertension. After stabilization with noninvasive ventilation and diuresis, the patient underwent minimally invasive surgical excision of the accessory membrane, resulting in complete relief of obstruction. Postoperative recovery was uneventful, with sustained clinical and echocardiographic improvement on follow-up.

**Discussion:**

This case illustrates the diagnostic challenge posed by CTS when presenting outside infancy and highlights the importance of early cardiac evaluation in pediatric patients with atypical respiratory presentations. Prompt use of bedside ultrasound and echocardiography enabled timely diagnosis and definitive management, leading to an excellent outcome.

**Conclusion:**

CTS, although rare, should be considered in children and adolescents presenting with unexplained pulmonary oedema or hypoxemia. Early recognition and surgical correction are associated with excellent short- and long-term outcomes.

## Introduction

Cor Triatriatum, Latin for “heart with three atria,” is a rare but surgically correctable congenital cardiac anomaly. It accounts for approximately 0.1% of all congenital heart defects (1). The left-atrial variant (cor Triatriatum sinister) is more common than the right-atrial form (cor Triatriatum dexter) (2).

**Figure 1:**
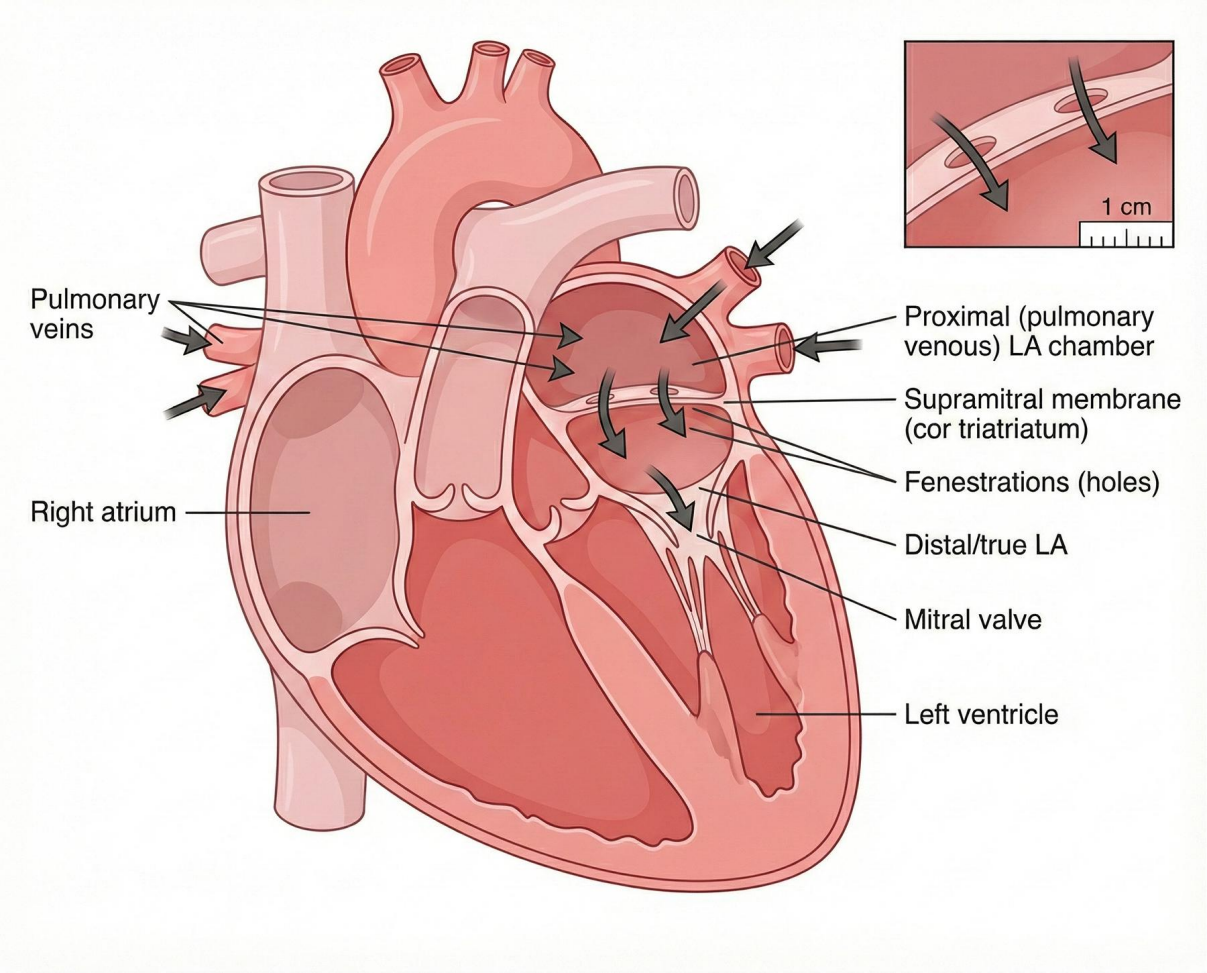
Schematic of Cor triatriatum sinister. The left atrium is divided by a supramitral fibromuscular membrane into a proximal pulmonary venous chamber and a distal true left atrium. Pulmonary veins drain into the proximal chamber; blood passes through fenestrations in the membrane into the true left atrium and then through the mitral valve to the left ventricle. Inset: magnified view of the membrane with fenestrations.

CTS is characterized by a fibromuscular membrane that subdivides the left atrium into a proximal pulmonary venous (accessory) chamber and a distal true left atrial chamber containing the left atrial appendage and communicating with the mitral valve (2, 3). The two chambers are connected by one or more fenestrations of variable size and number, which determine the degree of intra-atrial obstruction. (Figure 1) Depending on the severity of obstruction, impaired left ventricular inflow may occur, leading to elevated left atrial pressure and secondary pulmonary hypertension (2). Consequently, clinical presentation varies widely and is influenced by the size and number of fenestrations, the resulting intra-atrial pressure gradient, and the presence of associated congenital cardiac anomalies (1).

Several embryologic theories have been proposed to explain the formation of the dividing membrane, including the malincorporation, mal-septation, and entrapment theories. The most widely accepted is the malincorporation theory, in which failure of the common pulmonary vein to incorporate properly into the left atrium results in persistence of a septum-like structure. Other proposed mechanisms include entrapment of the common pulmonary vein by the left horn of the sinus venosus and abnormal malseptation of the atrial chamber (2). In this case, we present an adolescent patient with previously undiagnosed cor Triatriatum sinister who presented with acute decompensated heart failure and cardiogenic pulmonary edema, initially mimicking severe respiratory disease, highlighting the importance of early cardiac evaluation in atypical pediatric presentations.

## Case presentation

A 13-year-old previously healthy boy presented to the emergency department on 12 May 2025 with one week of progressive exertional dyspnea and dry cough, followed by an acute worsening of shortness of breath after swimming on the day of presentation. He reported dyspnea on exertion relieved by rest, without fever, sputum production, hemoptysis, chest pain, palpitations, or prior similar episodes. There was no personal or family history of cardiac or pulmonary disease.

**Figure 2:**
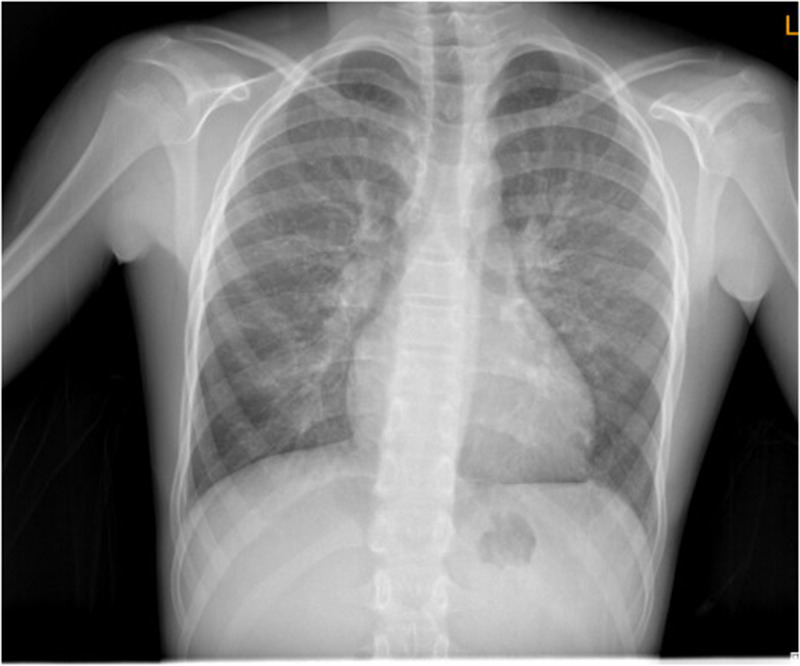
Posteroanterior (PA) chest radiograph demonstrating bilateral pulmonary infiltrates. The image shows diffuse opacities in both lung fields, consistent with pulmonary edema. Pulmonary vascular congestion is apparent. These findings correlate with the patient's acute hypoxic respiratory failure and were key in prompting further cardiac evaluation, including transthoracic echocardiography, which revealed cor triatriatum sinister. No focal consolidation or pleural effusion is noted, helping to differentiate cardiogenic pulmonary edema from primary respiratory infection.

On arrival, he was in severe respiratory distress with tachypnoea (respiratory rate 32–35/min), tachycardia (heart rate ∼150/min), and marked hypoxemia (oxygen saturation 67% on room air, improving to 92% with 8 L/min via non-rebreather mask). Blood pressure was 120/70 mmHg and temperature was normal. Physical examination revealed elevated jugular venous pressure and bilateral basal crackles on lung auscultation; no cardiac murmur was documented. Initial chest radiography demonstrated bilateral pulmonary congestion (Figure 2). Laboratory evaluation showed mild leukocytosis (WBC 12.8 × 10⁹/L) with low inflammatory markers (C-reactive protein 1 mg/L). Arterial blood gas analysis revealed respiratory alkalosis with severe hypoxemia (pH 7.43, PaCO₂ 29.8 mmHg, PaO₂ 40.4 mmHg, lactate 1.7 mmol/L).

Given acute hypoxic respiratory failure with radiographic pulmonary congestion and elevated JVP, a cardiogenic etiology was considered early in the intensive care unit. Point-of-care lung ultrasound demonstrated diffuse B-lines consistent with pulmonary oedema rather than focal consolidation. Focused transthoracic echocardiography raised suspicion for a left-sided obstructive intracardiac lesion. The patient showed rapid clinical improvement with non-invasive ventilatory support (BiPAP) and intravenous diuresis, further supporting a diagnosis of acute decompensated heart failure rather than primary pulmonary pathology.

**Figure 3:**
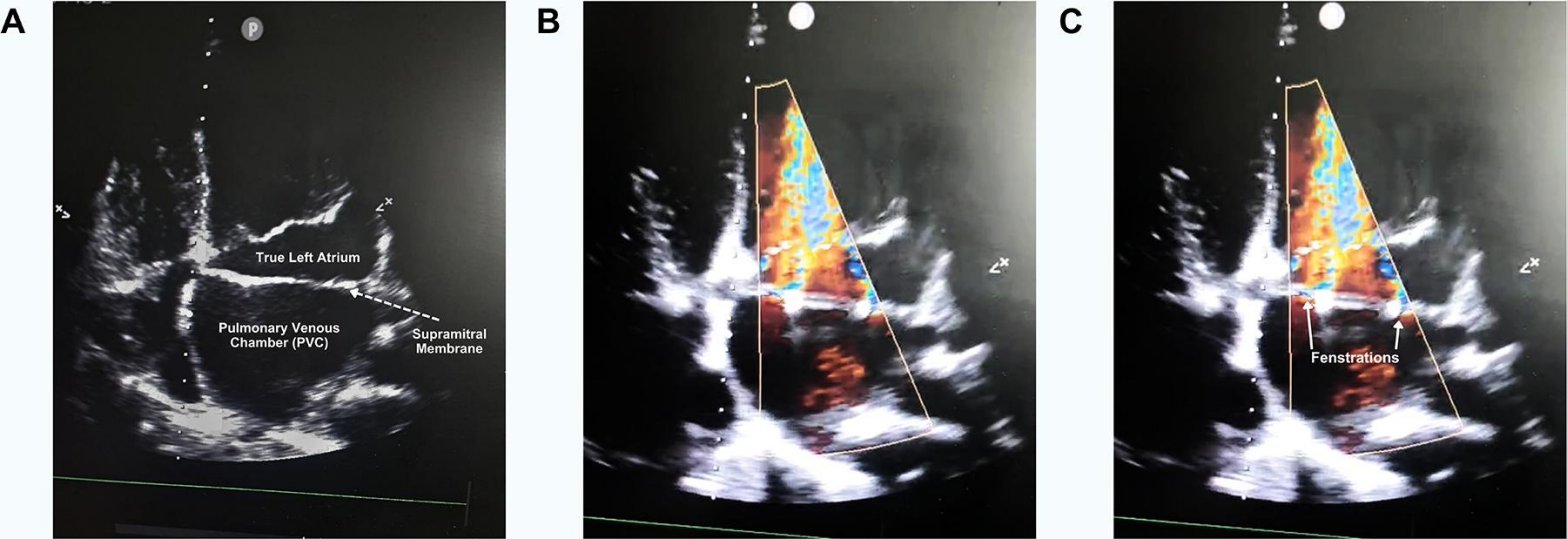
Apical four-chamber transthoracic echocardiographic images demonstrating Cor triatriatum sinister. **(A)** 2D apical four-chamber view showing a supramitral membrane that divides the left atrium into a posterior–superior pulmonary venous chamber (PVC) and an anterior–inferior true left atrium (LA); the true LA lies between the membrane and the mitral valve and communicates directly with the left ventricle (LV). **(B)** Color Doppler apical view demonstrating flow jets traversing multiple fenestrations in the membrane, indicating residual communication between the PVC and true LA. **(C)** Same color Doppler image as panel B, annotated with white arrowheads that mark the two principal fenestrations.

A comprehensive pediatric transthoracic echocardiogram revealed a supramitral fibromuscular membrane dividing the left atrium, consistent with cor Triatriatum sinister (Figure 3; Supplementary Video S1). Two fenestrations were identified within the membrane, with severe obstruction demonstrated by Doppler interrogation (peak gradient 25 mmHg, mean gradient 9 mmHg). There was trace tricuspid regurgitation with an estimated pulmonary artery systolic pressure of approximately 50 mmHg, indicating secondary pulmonary hypertension. Cardiac chamber dimensions and biventricular systolic function were otherwise normal.

In the setting of symptomatic pulmonary oedema, significant intra-atrial obstruction, and pulmonary hypertension, the patient was transferred to a pediatric cardiac surgery unit. Following preoperative assessment, including CT pulmonary angiography to delineate anatomy and exclude associated anomalies, he underwent surgical repair on 20 May 2025 consisting of minimally invasive circular resection of the supramitral membrane. Intraoperative transesophageal echocardiography confirmed complete relief of obstruction, absence of mitral stenosis, trivial mitral regurgitation, and preserved left ventricular systolic function.

Postoperative recovery in the cardiac care unit was uncomplicated. The patient was successfully weaned to room air, received a short course of milrinone, and was transitioned from intravenous to oral diuretic therapy. Serial transthoracic echocardiography prior to discharge demonstrated no residual membrane, trivial mitral regurgitation, and preserved systolic function (ejection fraction 58% on 25 May 2025, improving to 65% by 1 June 2025). He was discharged home on oral diuretics with planned outpatient cardiology follow-up.

At follow-up evaluation on 16 September 2025, the patient remained asymptomatic and was no longer receiving cardiac medications. Repeat echocardiography confirmed durable relief of obstruction, normal chamber sizes and ventricular function, and only trace tricuspid regurgitation with estimated pulmonary artery systolic pressure of 35–40 mmHg.

## Discussion

Cor Triatriatum sinister (CTS) is a rare congenital cardiac anomaly characterized by the presence of a fibromuscular membrane that subdivides the left atrium. Although frequently diagnosed in infancy, clinical presentation can be delayed and highly variable. Obstructive forms may present with pulmonary venous congestion that closely mimics primary respiratory pathology, contributing to diagnostic delay. Despite its rarity, CTS is surgically correctable, and long-term outcomes following definitive repair are generally excellent (4).

The clinical manifestations of CTS are primarily determined by the size of the orifice within the accessory atrial membrane. A small orifice produces significant obstruction, resulting in an intra-atrial pressure gradient that functionally resembles mitral stenosis, while symptom severity may also be influenced by associated congenital cardiac anomalies. In neonates and infants, the typically narrow communication leads to elevated proximal left atrial pressure and pulmonary venous congestion, with presentations ranging from mild respiratory distress to severe neonatal dyspnea associated with increased mortality risk (5).

In contrast, adults with CTS are often asymptomatic due to a larger membrane orifice and absence of a significant pressure gradient. When symptoms do occur later in life, they are usually related to progressive fibrosis or calcification of the membrane, resulting in delayed obstruction, although severe obstruction may occasionally present earlier (6). Adult manifestations include exertional dyspnea, orthopnea, hemoptysis, and, in rare cases, acute pulmonary oedema during labor in previously undiagnosed women (7, 8). CTS may also be revealed by atrial arrhythmias or cerebral and systemic embolic events secondary to thrombus formation within an enlarged accessory atrial chamber (6). On physical examination, auscultation may reveal a diastolic murmur, often accompanied by a loud P2 in the presence of pulmonary hypertension. The absence of an opening snap or accentuated S1 helps distinguish CTS from mitral stenosis, with murmur intensity depending on flow velocity across the membrane orifice (5, 6).

Echocardiography remains the diagnostic modality of choice for CTS, allowing detailed assessment of membrane morphology, transmembrane gradients, atrial and ventricular size and function, and associated cardiac lesions. Cross-sectional imaging with computed tomography or cardiac magnetic resonance imaging may be useful for surgical planning or in cases with suboptimal echocardiographic windows. In our patient, bedside echocardiography followed by formal pediatric transthoracic echocardiography established the diagnosis and severity of obstruction, facilitating timely referral for surgical management (4).

Management of CTS is guided by symptom burden and hemodynamic significance. Asymptomatic patients with incidental echocardiographic findings and no pressure gradient generally require no medical therapy. Symptomatic patients, particularly those with exertional dyspnea or pulmonary congestion, may benefit from medical management with diuretics, digoxin, and preload reduction; however, surgical intervention is typically indicated in this setting. Surgical correction is recommended for symptomatic children and adults with significant obstruction and involves excision of the accessory membrane, closure of associated atrial septal defects, and correction of anomalous venous return when present, following comprehensive preoperative imaging using modalities such as 3D transesophageal echocardiography, computed tomography, or cardiac magnetic resonance imaging. When performed in experienced centers, surgical outcomes are excellent, with most patients becoming asymptomatic and reported five-year survival rates exceeding 90% (4, 9). In our patient, acute decompensated heart failure with pulmonary oedema and echocardiographic evidence of severe supramitral obstruction prompted stabilization with noninvasive ventilation and diuresis, followed by definitive minimally invasive surgical excision of the accessory membrane with complete symptomatic and hemodynamic resolution.

A large retrospective series of 28 patients with CTS further highlights the early clinical manifestation and complexity of this condition. Most patients presented within the first year of life, with a median age at surgery of seven months. Lam type A variants, particularly subtype A1, were most common, while type B was rare and type C was not observed. Nearly half of the cohort had nonobstructive communications between atrial chambers, and more than half had associated congenital cardiac anomalies, several of which were hemodynamically significant. Common presenting features included tachypnoea, failure to thrive, and feeding difficulties, with a subset presenting in shock or respiratory arrest. Approximately one-fifth required emergency surgery within 24 h, whereas others underwent surgery during the initial admission or electively. These findings underscore the typically early and sometimes severe presentation of CTS (10).

This case highlights several important clinical considerations. First, cardiac etiologies should be included in the differential diagnosis of children and adolescents presenting with bilateral pulmonary opacities and disproportionate hypoxemia, particularly when inflammatory markers are low or jugular venous pressure is elevated. Second, early use of bedside cardiac and lung ultrasonography is invaluable in differentiating cardiogenic pulmonary edema from primary respiratory pathology. Third, prompt referral to pediatric cardiology is essential when intracardiac obstruction or elevated pulmonary pressures are suspected, as timely surgical intervention is associated with excellent outcomes. Although limited by its single-case design and relatively short hemodynamic follow-up, the favorable postoperative course observed in this patient is consistent with outcomes reported in published series (4).

## Conclusion

Cor Triatriatum sinister is an uncommon but surgically correctable congenital cardiac anomaly that may present beyond infancy with nonspecific respiratory symptoms, closely mimicking primary pulmonary disease. This case underscores the importance of maintaining a high index of suspicion for cardiac etiologies in pediatric patients presenting with bilateral pulmonary opacities, disproportionate hypoxemia, and low inflammatory markers. Early application of bedside cardiac and lung ultrasonography played a pivotal role in identifying a cardiogenic cause and expediting definitive management. Timely referral and minimally invasive surgical excision of the obstructive membrane resulted in complete symptomatic and hemodynamic resolution. Increased awareness of this rare entity may facilitate earlier diagnosis and improve outcomes in similar presentations.

## Data Availability

The original contributions presented in the study are included in the article/Supplementary Material, further inquiries can be directed to the corresponding author.

## Supplementary material

Supplementary Video S1 Focused transthoracic echocardiography demonstrating the obstructive supramitral membrane and fenestrations.
